# Biomechanical Impact of Cementation Technique Variations on Femoral Stem Stability: An In Vitro Polyurethane Model Study

**DOI:** 10.3390/jcm14103291

**Published:** 2025-05-08

**Authors:** Roland Fazakas, Laura Ioana Bondar, Csongor Toth, Brigitte Osser, Iosif Ilia, Caius Calin Miuta, Dan Fruja, Diana Carina Iovanovici, Liviu Gavrila-Ardelean, Alexandru Pop

**Affiliations:** 1 Doctoral School of Medicine, “Vasile Goldiș” Western University of Arad, 310025 Arad, Romania; fazakas.roland@uvvg.ro (R.F.); pop.alexandru@uvvg.ro (A.P.); 2 Department of Biology and Life Sciences, Faculty of Medicine, “Vasile Goldiș” Western University of Arad, 310025 Arad, Romania; 3 Doctoral School of Biomedical Sciences, University of Oradea, 410087 Oradea, Romania; csongor.toth@uav.ro (C.T.); brigitte.osser@uav.ro (B.O.); 4 Faculty of Physical Education and Sport, “Aurel Vlaicu” University of Arad, 310130 Arad, Romania; iosif.ilia@uav.ro (I.I.); caius.miuta@uav.ro (C.C.M.); 5 Department of General Medicine, Faculty of Medicine, “Vasile Goldiș” Western University of Arad, 310025 Arad, Romania; fruja.dan@uvvg.ro; 6 Institute of Cardiovascular Diseases Timișoara, Gheorghe Adam Street, No. 13A, 300310 Timisoara, Romania; iovanovici.dianacarina@student.uoradea.ro; 7 Prosthetic Dentistry, Faculty of Dental Medicine, “Vasile Goldiș” Western University of Arad, 310025 Arad, Romania; gavrila-ardelean.liviu@uvvg.ro

**Keywords:** biomechanical testing, cemented femoral stem, hip arthroplasty, in vitro study, overreaming, polyurethane bone surrogate, press-fit technique

## Abstract

**Background/Objectives:** Achieving optimal primary stability in cemented total hip arthroplasty remains a critical factor influencing long-term implant success. Variability in cementation techniques can significantly affect biomechanical performance, yet consensus on best practices is lacking. This study investigates the influence of cementation parameters on femoral stem fixation. **Methods:** This in vitro comparative study evaluated four cementation techniques—Classic (line-to-line), Press-Fit (undersized reaming), Overreaming (oversized reaming), and Valgus Malpositioning (15° deviation). An experimental model using standardized Polyurethane (PU) bone surrogates was developed. Mechanical testing assessed axial deformation and ultimate load capacity to failure. **Results:** The Press-Fit technique demonstrated significantly greater deformation (17.10 ± 0.89 mm) but a reduced load capacity (6317.47 ± 518.34 N) compared to the Classic approach. Overreaming and Valgus techniques both showed reduced mechanical performance, with Overreaming yielding the lowest structural integrity. **Conclusions:** Cement mantle thickness emerged as the primary determinant of biomechanical stability, surpassing the impact of implant positioning. While increased mantle thickness improves energy absorption, it may compromise ultimate strength. These findings underscore the importance of optimizing the cementation technique to balance flexibility and mechanical resistance, guiding surgical protocols toward improved implant longevity. This study introduces a novel integrative approach combining fluoroscopic assessment of cement mantle morphology with mechanical testing in a standardized model, providing new evidence on the relative influence of mantle thickness and implant malposition on femoral stem stability.

## 1. Introduction

Cemented hip arthroplasty has become one of the most frequently performed procedures for managing advanced hip osteoarthritis and other degenerative conditions, largely due to its ability to deliver immediate primary stability [1,2,3]. This stability is achieved through a secure implant–cement interface and a robust mechanical interlock with bone, ensuring effective load transfer during the critical postoperative period [4,5,6]. However, despite these benefits, the long-term durability of cemented implants is often compromised by the gradual mechanical deterioration of the cement mantle [7,8,9]. Factors such as microcracks, fatigue, and the accumulation of wear debris can lead to aseptic loosening—a significant cause of implant failure, particularly in younger and more active patients who impose higher mechanical demands on their prostheses [10,11,12,13].

Previous studies have underscored the importance of the cementation technique in influencing implant biomechanics [14,15,16]. Variations in the sizing relationship between the reamer and the implant, along with differences in cement mantle thickness and homogeneity, have been shown to affect stress distribution at both the implant–cement and cement–bone interfaces [17,18,19]. Despite these insights, there remains considerable debate regarding the optimal cementation configuration to maximize both stability and longevity [20,21,22]. These divergent views highlight the complexity of achieving an ideal balance between a durable fixation and minimizing long-term complications [23,24].

Given the conflicting evidence in the literature and the clinical challenges associated with cement mantle deterioration, there is a clear need for a standardized, reproducible method to evaluate the mechanical performance of various cementation techniques. This study aims to address this gap by developing an in vitro model that simulates the biomechanical environment of a cemented femoral stem. By systematically altering cementation parameters—specifically, cement mantle thickness and implant positioning—it is intended to quantify the migration and deformation behavior of the implant. The insights gained from this investigation are expected to provide a more comprehensive understanding of how cementation technique influences primary stability, ultimately guiding improvements in surgical practice and patient outcomes.

In summary, this study provides the first comparative biomechanical analysis integrating cement mantle thickness variations and implant malposition in a standardized Polyurethane (PU) bone model. By correlating fluoroscopic imaging with mechanical testing, it clarifies the combined effects of reaming technique and implant alignment on primary stem stability, offering clinically relevant insights to optimize cementation strategy.

## 2. Materials and Methods

### 2.1. Specimen Preparation

To minimize the variability inherent in cadaveric bone, PU foam was selected as a bone surrogate. The PU foam, with a density of 0.35 g/cm^3^ (ASTM grade 20 [25]), exhibits mechanical properties comparable to those of normal femoral and pelvic bone. Cylindrical specimens were fabricated with a diameter of 50 mm and a length of 200 mm. One end of each specimen was beveled at a 45° angle to simulate an osteotomy and a central intramedullary canal measuring 8.5 mm in diameter and 160 mm in depth was created to facilitate cement compression.

**Figure 1:**
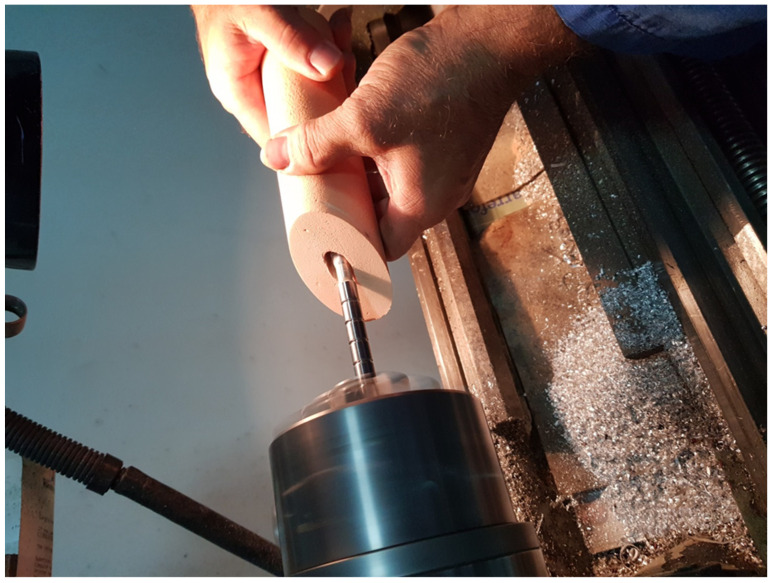
Creation of the intramedullary canal in PU foam specimens to simulate the femoral canal.

The specimen preparation process is illustrated in Figure 1, showing the creation of the intramedullary canal. This step was essential to ensure proper cement interdigitation and uniform cement mantle formation around the femoral stem. The precision of canal formation contributed to the reproducibility of the mechanical testing conditions used in this study.

For each cementation technique, 30 specimens were prepared. PU foam blocks were used as synthetic bone surrogates to ensure consistent and reproducible testing conditions [25,26]. This approach aligns with current international guidelines for the mechanical testing of orthopedic implants. Specifically, the selected foam meets the criteria outlined in ASTM F1839—Standard Specification for Rigid Polyurethane Foam for Use as a Standard Material for Testing Orthopaedic Devices and Instruments [27]. This standard defines acceptable density and mechanical properties for simulating cancellous bone. The use of standardized PU foam also ensures compatibility with ISO 7206-4 [28] and ISO 7206-6 [29], which govern mechanical testing of femoral stem implants under axial and torsional loads. This standardized methodology enables controlled evaluation of cementation techniques, independent of the anatomical variability found in cadaveric specimens [30,31,32].

Randomization and blinding were not applicable in this study, as all specimens were prepared and tested under standardized conditions using identical procedures to minimize variability. The sample size (*n* = 30 per group) was selected based on prior similar biomechanical studies to ensure sufficient statistical power for detecting differences in mechanical performance.

Detailed cutting and machining protocols, as well as a complete list of materials, are provided in Supplementary File S1 to ensure reproducibility. This in vitro study was designed and reported in accordance with the CRIS (Checklist for Reporting In Vitro Studies) guidelines (Supplementary File S2).

### 2.2. Implant and Cementation Technique

A Zimmer™ (Zimmer Biomet, Warsaw, IN, USA) Metabloc femoral stem (126 mm in length, featuring a three-dimensional conical design with proximal fixation) was employed. Four distinct cementation techniques were evaluated using the same number of specimens (n = 30 per group). A priori sample size estimation was performed using G*Power (version 3.1.9.7, Heinrich Heine Universität Düsseldorf, Düsseldorf, Germany) for a one-way ANOVA with four groups, assuming an alpha of 0.05, power of 80%, and a large effect size (f = 0.40) based on previous biomechanical studies. The analysis indicated a minimum required sample size of 12 specimens per group; therefore, 30 specimens per group were used to ensure sufficient statistical power and reproducibility. The first technique, referred to as Classic (Line-to-Line), involved reaming the femoral canal with a rasp corresponding exactly to the implant size. The second technique, known as Press-Fit, utilized an undersized reamer, which produced a thinner cement mantle due to the reduced space between the implant and the canal wall. The third technique, termed Overreaming, was performed using an oversized reamer, resulting in a thicker cement mantle created by the increased canal space. The fourth method involved Valgus Malpositioning, in which the implant was inserted at a 15° valgus orientation to simulate non-anatomical placement.

Cementation was performed using Polymethyl Methacrylate (PMMA) bone cement, mixed, and applied strictly according to the manufacturer’s instructions. Silicone tubes were used to evacuate trapped air and promote uniform cement distribution. In the Valgus group, a dedicated alignment device was used to maintain the 15° deviation during the curing process.

**Figure 2:**
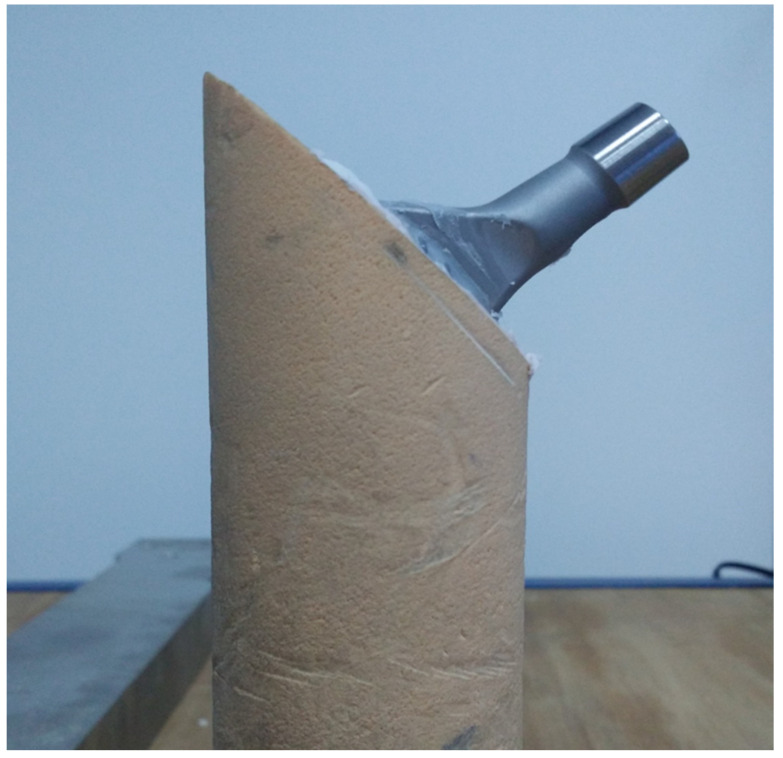
Cemented femoral stem in bone surrogates.

Following cementation, the final integration of the femoral stems within the PU bone surrogates is shown in Figure 2, illustrating differences in cement mantle thickness and implant alignment.

All specimens were allowed to cure at room temperature (22 ± 2 °C) and 40–50% relative humidity for 24 h prior to mechanical testing.

### 2.3. Cement Mantle Thickness Evaluation

To visually highlight the differences in cement mantle morphology among the four cementation techniques, representative specimens from the Classic (Line-to-Line), Press-Fit (Undersized), Overreaming (Oversized), and Valgus Malpositioning groups were selected and fluoroscopically imaged under standardized conditions. The distance between the X-ray source and the specimens was held constant throughout, with all prostheses positioned on a radiolucent table. No modifications were made to the irradiation distance or equipment height across groups.

Pixel-based calibration confirmed that 1 pixel corresponds to 0.37 mm, enabling an accurate assessment of cement mantle thickness.

**Figure 3:**
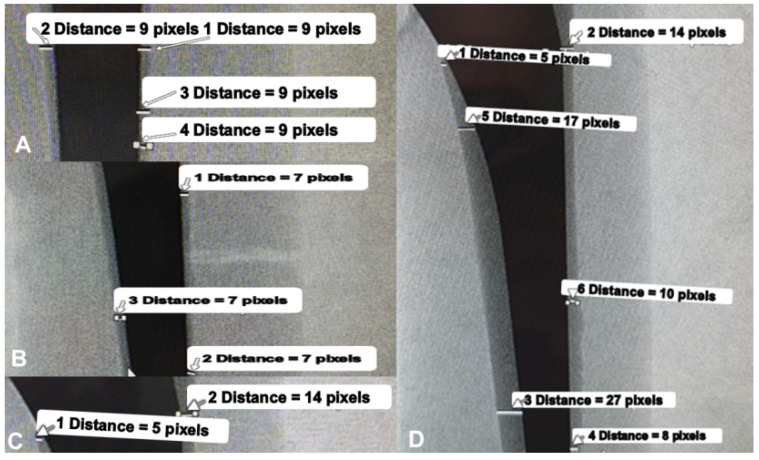
Fluoroscopic images of representative specimens implanted using different cementation techniques: (**A**) Classic (Line-to-Line), (**B**) Press-Fit (Undersized), (**C**) Overreaming (Oversized), and (**D**) Valgus Malpositioning.

In the Classic (Line-to-Line) group, the cement mantle appeared symmetrical and uniform, averaging 9 pixels (~3.33 mm). This value falls within the clinically recommended range of 3–5 mm, supporting the technique’s suitability for consistent cement distribution (Figure 3A).

The Press-Fit method produced a thinner and less uniform cement mantle, averaging 7 pixels (~2.59 mm). While still acceptable, the reduced thickness may suggest a limited capacity for optimal stress distribution (Figure 3B).

In the Overreaming group, the use of an oversized rasp led to the outward displacement of the prosthesis. This resulted in an asymmetrical mantle, with the internal layer reaching 14 pixels (~5.18 mm)—exceeding ideal limits and potentially increasing brittleness and the risk of microcracking (Figure 3C).

The Valgus Malpositioning configuration showed the most significant asymmetry. The cement mantle varied considerably along the stem, reaching 27 pixels (~9.99 mm) on the medial side and tapering to 8 pixels (~2.96 mm) laterally. This pronounced reversal in distribution demonstrates a clear deviation from anatomical alignment, likely resulting from intentional malpositioning during cementation (Figure 3D).

These findings confirm that each technique yields a distinct cement mantle profile, likely influencing biomechanical performance under load. Among the four groups, the Valgus Malpositioning technique demonstrated the greatest discrepancy in cement mantle thickness, while the Classic method exhibited the most uniform distribution, aligning closely with clinical recommendations.

### 2.4. Mechanical Testing

Mechanical testing was performed using a bi-axial servo-hydraulic Instron™ 8874 testing machine (Illinois Tool Works Inc., Norwood, MA, USA), equipped with a custom-designed fixture to ensure rigid fixation of the specimens. A 150 mm diameter compression plate was utilized to apply axial load directly onto the femoral head. Prior to testing, both the machine and the fixture were calibrated according to the manufacturer’s recommendations.

All tests were carried out under controlled laboratory conditions (22 ± 2 °C; 40–50% relative humidity) at a constant uniaxial displacement rate of 5 mm/min. Each test was continued until one of three endpoints was reached: either a total displacement of 20 mm, a peak load of 11,000 N, or a visible fracture of the construct.

These loading parameters were selected to reflect peak in vivo forces typically encountered during high-demand activities, such as stair ascent and descent, where hip joint loads can reach up to 4200 N.

**Figure 4:**
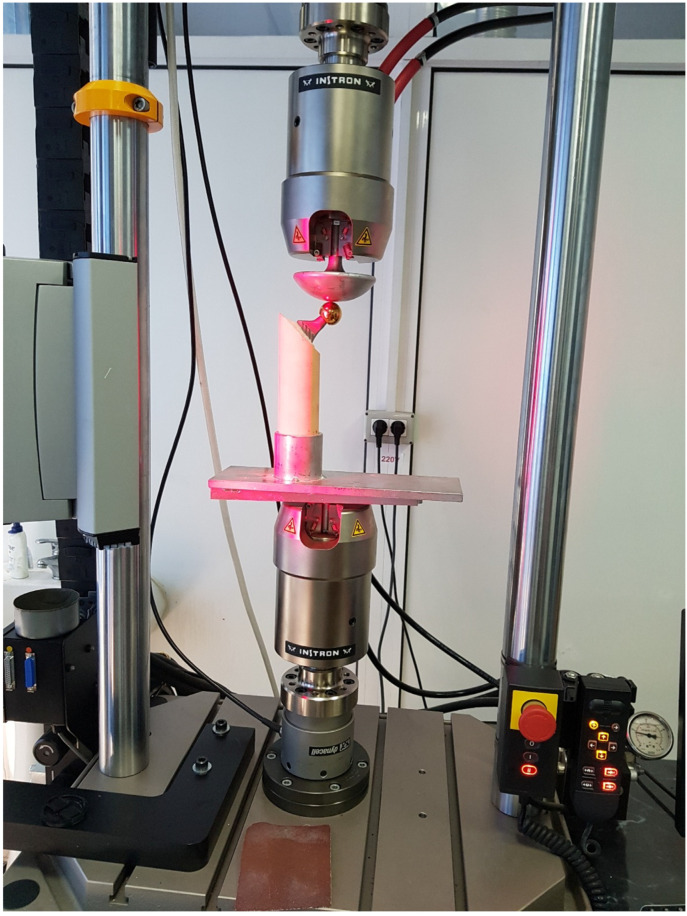
Experimental setup for axial mechanical testing using the Instron™ 8874 machine.

A visual of the mechanical testing setup is presented in Figure 4, showing the mounted femoral stem specimen under load application.

### 2.5. Data Analysis

Mechanical testing data were analyzed to evaluate the performance of femoral stem fixation across four cementation techniques: Classic, Press-Fit, Overreaming, and Valgus Malpositioning. Two primary mechanical parameters were extracted from the load-deformation curves: compression at break (mm) and ultimate load at break (N).

Data are reported as mean ± standard deviation (SD). Statistical comparisons between groups were performed using one-way analysis of variance (ANOVA) followed by Tukey’s post hoc test for pairwise analysis. A *p*-value < 0.05 was considered statistically significant. All analyses were conducted using IBM SPSS Statistics, Version 26.0 (IBM Corp., Armonk, NY, USA).

Compression at break was defined as the total displacement from the initial position (L_0_) to the point of failure, corresponding to the maximum load. Load at break was defined as the peak force recorded during testing. These values were extracted directly from the load-displacement curves using ZwickRoell testXpert III software (ZwickRoell GmbH & Co. KG, Ulm, Germany), with a manual review for accuracy.

Each group included 30 specimens, resulting in a total of 120 tested configurations. All raw data and analytical calculations are available from the corresponding author upon reasonable request.

### 2.6. Hypotheses of the Study

The aim of this pilot study is to evaluate how different cementation techniques affect the primary stability of cemented femoral stems. To guide this investigation, the following hypotheses were developed based on existing literature and preliminary observations:

1. Press-Fit Technique: Using an undersized reamer to create a thinner cement mantle will enhance the deformation capacity of the femoral stem compared to the Classic (Line-to-Line) technique.
2. Ultimate Load Capacity: Despite the increased deformation capacity, the Press-Fit technique may yield a lower ultimate load capacity than the Classic method due to potential issues with cement mantle uniformity and stress concentration.
3. Overreaming Technique: Reaming with an oversized tool, resulting in a thicker cement layer, will lead to reduced mechanical stability, as reflected by lower deformation and load capacity compared to the Classic technique.
4. Valgus Malpositioning: Simulating non-anatomical implant placement by inserting the stem at a 15° valgus orientation will further compromise primary stability, evidenced by decreased load capacity and increased implant migration.

## 3. Results

### 3.1. Overall Overview

The load-deformation curves obtained for all cementation techniques exhibited two distinct phases. The initial linear elastic phase demonstrated proportional deformation with increasing load, followed by a plastic phase characterized by irreversible deformation and progressive structural failure. All specimens sustained loads exceeding physiological thresholds encountered during daily activities, such as stair ascent and descent (~4200 N).

Key mechanical parameters are summarized in Table 1, including mean values, standard deviations, and statistical comparisons relative to the Classic technique.

The Press-Fit technique exhibited significantly greater deformation capacity (17.10 ± 0.89 mm, *p* < 0.01) compared to Classic (14.56 ± 1.12 mm), but significantly lower load-bearing capacity (6317.47 ± 518.34 N vs. 10,330.23 ± 634.11 N, *p* < 0.01), indicating increased flexibility at the expense of strength.

The Overreaming group showed significantly lower values for both deformation (12.23 ± 1.03 mm, *p* < 0.05) and load at break (9454.76 ± 583.22 N, *p* < 0.05), suggesting reduced ductility and structural integrity.

The Valgus group demonstrated a significant reduction in deformation (11.77 ± 1.15 mm, *p* < 0.05), while the difference in load-bearing capacity (9695.51 ± 561.47 N) did not reach statistical significance (*p* = 0.08), indicating that valgus malalignment primarily affects flexibility.

These findings support the interpretation that cement mantle thickness, governed by the cementation technique, exerts a more substantial influence on mechanical performance than implant positioning alone.

**Figure 5:**
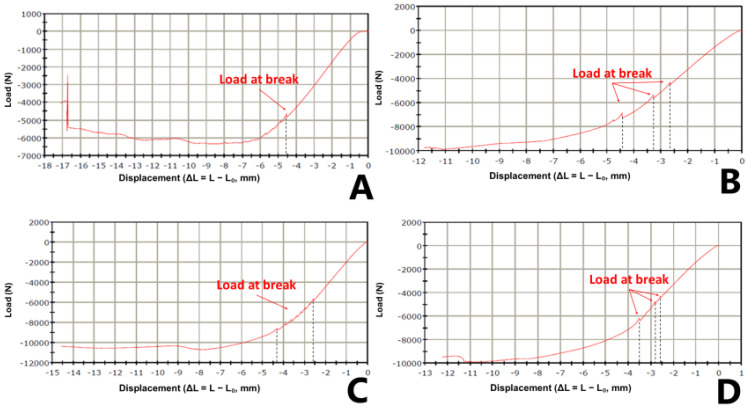
Load–displacement curves for the different cementation techniques: (**A**) Press-Fit (Undersized), (**B**) Classic (Line-to-Line), (**C**) Valgus Malpositioning, and (**D**) Overreaming (Oversized). The point of “Load at break” is marked for each curve, representing the ultimate load before failure.

These quantitative findings presented in Table 1 are further supported by the corresponding load-displacement curves shown in Figure 5, which visually illustrate the mechanical behavior of each cementation technique.

### 3.2. Press-Fit Technique Performance

The Press-Fit cementation technique, which involved the use of an undersized reamer, resulted in the highest deformation capacity among all tested groups. This technique produced a thinner and less uniform cement mantle, as confirmed by imaging, averaging 7 pixels (~2.59 mm). The average displacement at break was 17.10 ± 0.89 mm, representing a 17.4% increase compared to the Classic technique (14.56 ± 1.12 mm) and a 39.9% increase compared to the Overreaming group (12.23 ± 1.03 mm).

This substantial increase in deformation suggests that, despite the thinner cement layer, the mantle’s material properties allowed for greater energy absorption under axial loading. However, this gain in flexibility came at the expense of reduced ultimate load capacity. The average load at the break for the Press-Fit group was significantly lower than that of the Classic group (6317.47 ± 518.34 N vs. 10,330.23 ± 634.11 N, *p* < 0.01), indicating a mechanical trade-off between deformability and strength.

The load-displacement curve for the Press-Fit group, shown in Figure 5A, illustrates this behavior. The *x*-axis displays negative displacement values, consistent with the compression testing convention (ΔL = L − L₀). The curve demonstrates an extended deformation range, peaking at approximately −1 mm, which corresponds to the point of maximum load before failure—clearly marked as “Load at break”. This behavior visually confirms the data presented in Table 1.

### 3.3. Ultimate Load Capacity Comparison

Although the Press-Fit technique demonstrated superior deformation capacity, it exhibited the lowest ultimate load capacity among all tested configurations. The Classic group achieved the highest mean load at break (10,330.23 ± 634.11 N), followed by the Valgus (9695.51 ± 561.47 N) and Overreaming (9454.76 ± 583.22 N) groups. In contrast, the Press-Fit group reached only 6317.47 ± 518.34 N, representing a 38.8% reduction compared to the Classic technique.

These findings underscore a mechanical trade-off between flexibility and strength. While a thinner cement mantle, as produced by the Press-Fit technique, may enhance the implant’s ability to absorb deformation under load, it can also compromise structural integrity. This is likely due to non-uniform cement distribution or increased internal stress within the mantle, both of which may reduce overall load-bearing capacity.

The characteristic load-displacement responses for each cementation technique are illustrated in Figure 5A–D. Figure 5A shows the Press-Fit technique, demonstrating increased deformation but reduced ultimate load. Figure 5B presents the Classic technique, which shows the highest load-bearing performance with a moderately extended deformation range. Figure 5C illustrates the Valgus malpositioning group, revealing slightly reduced deformation and a comparable, though marginally lower, ultimate load capacity. Figure 5D shows the Overreaming technique, characterized by a thicker cement mantle and reduced mechanical resistance.

Despite its reduced thickness, the Press-Fit mantle exhibited greater deformation capacity, highlighting a complex relationship between mantle geometry and mechanical behavior.

### 3.4. Overreaming Technique Effects

The Overreaming technique, which produced a thicker cement mantle through the use of an oversized reamer, led to a modest reduction in both deformation capacity and ultimate load capacity compared to the Classic approach. These findings confirm that increased mantle thickness can compromise mechanical stability by altering the cement’s ability to distribute load uniformly, thereby increasing stress concentrations within the cement layer and at the bone-implant interface.

**Figure 6:**
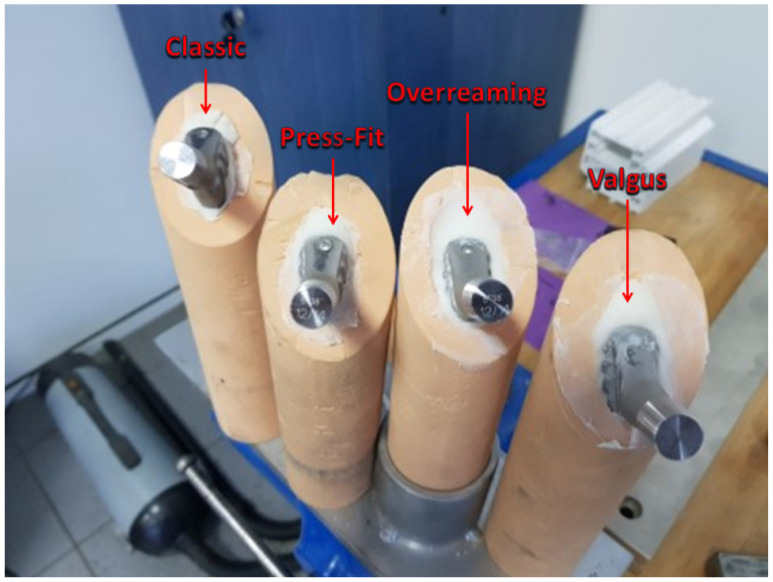
Differences in cement mantle thickness between cementation techniques.

Differences in cement mantle thickness among the tested cementation techniques are clearly illustrated in Figure 6. In this image, the overreamed configuration shows a visibly thicker cement layer surrounding the implant compared to the Classic and Press-Fit techniques. The femoral stem appears more centered within the polyurethane block, confirming a wider cement buffer. This structural difference aligns with the mechanical trends observed in this study, where increased mantle thickness led to decreased load-bearing capacity and a higher risk of brittle fracture or early failure.

### 3.5. Valgus Malpositioning Impact

In the valgus malpositioning group, the implant was inserted at a 15° valgus angle to simulate non-anatomical placement. Although the ultimate load capacity in this group (approximately 9695 N) was comparable to that of the classic technique, the deformation performance was slightly compromised.

**Figure 7:**
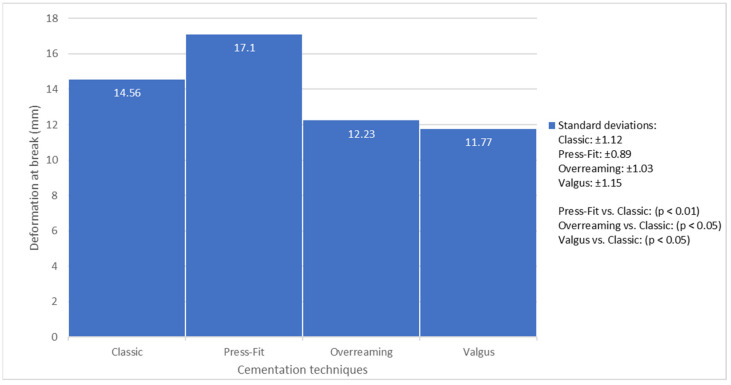
Comparison of deformation at break for different cementation techniques.

While valgus malpositioning does affect load distribution, the findings indicate that cement mantle thickness plays a more dominant role in determining mechanical stability and ultimate load capacity. These results are presented in Figure 7.

## 4. Discussion

This study investigated how cementation technique and implant positioning affect the mechanical performance of cemented femoral stems, evaluating deformation capacity and ultimate load resistance. The Press-Fit technique produced the greatest deformation but the lowest ultimate load capacity, indicating increased flexibility at the expense of strength. The Overreaming technique resulted in reduced deformation and lower load capacity compared to the Classic technique. Valgus malpositioning produced similar load capacity but slightly reduced deformation. Overall, cement mantle thickness emerged as the primary factor influencing implant stability, with thinner mantles increasing deformation but compromising strength, while malpositioning had a lesser impact than anticipated.

### 4.1. Press-Fit Cementation: Increased Deformation but Reduced Load Capacity

The Press-Fit technique, which utilized an undersized reamer and resulted in a thinner cement mantle, demonstrated improved deformation capacity compared to the Classic and Overreaming techniques. This suggests that, despite the reduced thickness, the cement layer was able to absorb more energy under load—potentially due to differences in material behavior or stress transfer at the implant–cement interface.

However, this benefit in flexibility was accompanied by a noticeable reduction in ultimate load capacity. This reduction can be attributed to non-uniform cement mantle formation or incomplete interdigitation of cement within the femoral canal. Additionally, it may be explained by stress distribution patterns and interfacial mechanics. Undersized reaming reduces cement mantle thickness, which may introduce localized stress concentrations within the cement layer. These regions can act as initiation sites for microcracks, particularly under compressive loading. While a thicker mantle can reduce these stress concentrations, it may also decrease the overall stiffness of the stem–bone composite, potentially increasing the risk of bulk cement failure or debonding at the cement–stem interface. These mechanisms align with previous finite element analyses and experimental findings that highlight the trade-off between increased deformability and decreased structural integrity in cemented fixation systems [33,34,35]. Additionally, clinical studies have highlighted the significance of identifying temporal patterns in complications post-arthroplasty, further reinforcing the need to optimize cementation techniques to prevent long-term implant failure [36,37].

Therefore, although the Press-Fit approach may be beneficial in terms of short-term flexibility and energy absorption, its reduced load-bearing performance raises concerns regarding long-term fixation and potential failure under repetitive or high-stress physiological conditions [38,39,40]. Further computational modeling could provide additional insight into stress transfer and crack propagation dynamics.

Although the mantle was thinner, its behavior under load suggests that mantle geometry and interfacial bonding may play a more significant role in deformation capacity than thickness alone.

### 4.2. Overreaming: Reduced Deformation and Stability

The Overreaming technique, which produced a thicker cement mantle due to the use of an oversized reamer, resulted in both lower deformation capacity and reduced ultimate load resistance compared to the Classic technique. This diminished mechanical performance can be explained by compromised load distribution within the cement layer, leading to increased stress transmission to the bone–cement interface.

While overreaming is sometimes employed to enhance implant fixation by increasing cement volume, these results suggest that excessive mantle thickness may weaken structural integrity—potentially increasing the risk of early failure due to brittle fracture or microcracking within the cement layer. Thus, achieving an optimal cement mantle thickness remains critical to balancing implant stability and effective stress dissipation [41,42,43].

### 4.3. Valgus Malpositioning: Moderate Impact on Load Capacity

Contrary to initial hypotheses, the valgus malpositioning group exhibited an ultimate load capacity (9695 N) comparable to that of the classic technique (10,330 N). While malpositioning can alter load transmission within the cement mantle, the findings suggest that a well-distributed cement layer can compensate for deviations in implant alignment.

The slight decrease in deformation capacity observed in the valgus group indicates that improper positioning may still contribute to uneven load distribution, potentially increasing stress at localized areas of the cement mantle. However, compared to the press-fit and overreaming techniques, valgus malpositioning had a less pronounced effect on mechanical performance.

These results highlight the importance of precise implant alignment, but they also suggest that achieving proper cement mantle thickness may be more influential in maintaining implant stability than minor deviations in implant positioning [44,45].

### 4.4. Clinical Implications and Future Directions

The results of this study reinforce the critical role of cement mantle configuration in optimizing biomechanical performance and ensuring long-term implant stability. While thinner cement mantles, as observed in the Press-Fit technique, enhanced deformation capacity, they also compromised ultimate load-bearing strength—likely due to non-uniform cement distribution and increased stress concentrations. Conversely, thicker cement mantles, as seen in the Overreaming technique, were associated with reduced mechanical performance, potentially due to increased brittleness and susceptibility to microcracks within the bulk cement. These issues may accelerate implant loosening or failure under physiological loads.

From a surgical perspective, these findings suggest that cementation strategies should aim for an optimal balance—neither excessively thick nor excessively thin—to maximize primary stability while ensuring long-term durability. The selection of an appropriate cementation technique should take into account patient-specific factors such as bone quality, activity level, and implant design. Older patients with osteoporotic bone may benefit from a moderately thicker cement mantle to enhance load distribution, whereas younger, more active patients may require a slightly thinner but more uniform mantle to withstand higher cyclic loads. Highly active individuals place greater mechanical demands on cemented implants, increasing the risk of cement fatigue failure. This may necessitate modifications to cement composition or alternative fixation methods, such as hybrid or uncemented approaches. Variations in stem geometry and surface characteristics also influence how cement interlocks with the implant, affecting long-term fixation and resistance to micromotion.

To further explore these biomechanical findings, future research should focus on long-term fatigue testing to assess how cyclic loading and repetitive mechanical stresses influence cement mantle degradation over time. This would help evaluate failure mechanisms related to microcracking and cement fragmentation under physiological conditions. Computational modeling, particularly finite element analysis, could provide a deeper understanding of stress distribution within the cement mantle under various loading conditions and implant alignments, predicting implant failure risk and optimizing cementation techniques. Clinical validation through retrospective and prospective studies would be necessary to determine whether the trends observed in vitro translate into actual clinical outcomes, including implant survivorship, revision rates, and patient-reported functional outcomes.

Further investigations into cement modification strategies may provide insights into improving mechanical properties and reducing long-term implant failure rates. Research into antibiotic-loaded cement and fiber-reinforced composites could help enhance cement strength and longevity. Additionally, advancements in surgical precision, such as robot-assisted and AI-guided cementation techniques, could ensure optimal cement mantle thickness and implant alignment, further improving the success of cemented femoral stem fixation.

This study underscores the importance of optimizing cementation techniques to enhance femoral stem fixation and longevity. While press-fit cementation increases deformation capacity, it also presents potential mechanical trade-offs that must be carefully considered in clinical practice. Future advancements in fatigue testing, computational modeling, and surgical technology could lead to refined cementation strategies, improved implant survivorship, and better patient outcomes in total hip arthroplasty.

Unlike prior studies evaluating cement mantle thickness or malposition separately, this study uniquely integrates both variables in a single biomechanical framework, correlating fluoroscopic imaging with mechanical testing to clarify their combined impact on femoral stem fixation.

### 4.5. Limitations of the Study

While this study provides valuable insights into the biomechanical behavior of cemented femoral stems under varying cementation techniques, several limitations should be acknowledged to contextualize the findings and guide future research.

First, the in vitro nature of the study is a key limitation. Although synthetic PU bone surrogates ensured standardization and reproducibility, they do not fully replicate the viscoelastic properties, structural heterogeneity, or remodeling capacity of human bone. Therefore, cadaveric validation or in vivo clinical studies are necessary to confirm the translational applicability of these results.

Second, the study was conducted under static axial loading conditions, without incorporating cyclic or fatigue testing. This setup does not reflect the repetitive, multidirectional forces encountered in vivo during daily activities such as walking or stair climbing. As such, the long-term performance and degradation of the cement–implant interface remain unassessed. Future studies should include dynamic testing protocols to evaluate fatigue behavior and cement durability under physiological conditions.

Third, the implant malpositioning model was simplified, simulating only a 15° valgus deviation. In clinical scenarios, additional variables such as anteversion, patient-specific bone geometry, and asymmetrical cement mantles may further influence fixation outcomes. More complex models incorporating multidirectional malalignment and anatomical variability would improve the clinical relevance of biomechanical assessments.

The relatively modest sample size may also limit statistical power and generalizability. While sufficient for initial comparisons, larger-scale, multi-center studies are needed to validate the results across diverse patient populations and implant designs.

Additionally, the study does not account for biological factors such as bone remodeling, osteolysis from wear debris, or biochemical interactions at the cement–bone interface. Environmental factors such as temperature variation and fluid exposure—common in vivo—may also affect cement behavior and mechanical integrity. Longitudinal in vivo models or advanced simulations incorporating these variables will be essential for comprehensive evaluation.

Finally, although focusing on four cementation techniques under static loading provided methodological clarity, this does not capture the full spectrum of real-world fixation strategies. Future research should explore alternative cement types, hybrid fixation approaches, and a broader range of biomechanical conditions to enhance our understanding of implant performance.

Despite these limitations, this study offers foundational evidence on the biomechanical implications of cement mantle configuration and implant alignment. Addressing these limitations through cadaveric validation, fatigue testing, and biologically integrated models will be essential to advancing clinical translation and improving outcomes in total hip arthroplasty.

## 5. Conclusions

This study highlights the critical role of cement mantle thickness in the biomechanical performance of cemented femoral stems. Variations in cementation technique, particularly mantle thickness, and implant positioning, directly impacted deformation capacity and load-bearing strength.

The Press-Fit technique increased deformation but reduced ultimate load capacity, while the Overreaming technique lowered both deformation and strength. Valgus malpositioning had a moderate effect on load capacity but showed that cement mantle thickness was a more dominant factor than implant alignment.

Achieving optimal cement mantle thickness is essential to balance implant stability and longevity. A mantle that is too thin or too thick may compromise mechanical integrity. These findings support the need for precise cementation techniques to optimize outcomes in total hip arthroplasty.

Future research should include long-term fatigue testing, computational modeling, and clinical validation to further assess the impact of cementation techniques on implant survival.

## Figures and Tables

**Table 1:** Comparative mechanical parameters for different cementation techniques of femoral stem implantation.

Cementation Technique	Compression at Break (mm)	Load at Break (N)	*p* (Compression)	*p* (Load)
Classic	14.56 ± 1.12	10,330.23 ± 634.11	—	—
Press-Fit	17.10 ± 0.89	6317.47 ± 518.34	*p* < 0.01	*p* < 0.01
Overreaming	12.23 ± 1.03	9454.76 ± 583.22	*p* < 0.05	*p* < 0.05
Valgus	11.77 ± 1.15	9695.51 ± 561.47	*p* < 0.05	*p* = 0.08

Note: Classic group used as the reference for all statistical comparisons.

## Data Availability

The data supporting the findings of this study are available from the corresponding author upon reasonable request.

## Supplementary Materials

The following supporting information can be downloaded at: https://www.mdpi.com/article/10.3390/jcm14103291/s1, File S1: Detailed Protocols and Materials. File S2. Checklist for Reporting In-vitro Studies (CRIS).

## Abbreviations

The following abbreviations are used in this manuscript:

PU	Polyurethane
PMMA	Polymethyl Methacrylate
CRIS	Checklist for Reporting In Vitro Studies
