# Supplementary material for: Biomechanical Impact of Cementation Technique Variations on Femoral Stem Stability: An In Vitro Polyurethane Model Study
Source: J Clin Med. 2025 May 8;14(10):3291. doi: 10.3390/jcm14103291 (PMC12112234; doi:10.3390/jcm14103291)
Supplement: Supplementary file 1 [file jcm-14-03291-s001.zip › File S1 - Detailed Protocols and Materials.pdf]

Supplementary File S1. Detailed Protocols and Materials

1. Specimen Preparation Protocol

1.1. Polyurethane (PU) Foam Block Preparation

- Material: Polyurethane foam, ASTM Grade 20, density 0.35 g/cm³ (Sawbones, USA; Catalog No. 1522-05).
- Block dimensions: 50 mm diameter × 200 mm length.
- Cutting procedure:
    - A band saw (Model: Struers Accutom-50, Struers A/S, Denmark) was used with a fine-tooth blade (TPI: 18) at a speed of 2000 RPM.
    - A 45° bevel osteotomy was created at one end using a rotary tilting jig.
    - Cutting tolerance: ±0.2 mm.

1.2. Intramedullary Canal Machining

- Canal diameter: 8.5 mm.
- Machining equipment: Proxxon TBM 220 precision bench drill with a solid carbide twist drill bit (8.5 mm diameter, Dormer Tools).
- Drilling speed: 1,500 RPM.
- Drilling depth: 160 mm, measured using a depth gauge.
- Cooling: Dry drilling without coolant.
- Debris removal: Compressed air cleaning after drilling.

2. Cementation Procedure

2.1. Femoral Stem

- Implant model: Zimmer™ Metabloc femoral stem (126 mm length; standard neck).
- Surface finish: Polished, 3D conical design.

2.2. Cement

- Material: Palacos® R+G bone cement (Heraeus Medical GmbH, Germany; Lot No. 347621A).
- Mixing method:
    - Vacuum mixing in Heraeus VACUUM MIXER (Model: VACUUM MIX 1).
    - Mixing duration: 45 seconds at vacuum pressure of 650 mmHg.
    - Cement was injected 2 minutes after mixing onset.

2.3. Cement Application

- Silicone tubing (Ø 4 mm) inserted distally to allow air evacuation.
- Cement pressurized manually using a cement gun (Zimmer® Cement Gun 2) at a constant manual force.
- Implant insertion performed by hand using a positioning jig for Classic and Press-Fit groups; valgus alignment achieved using a custom 15° wedge jig.
- Curing conditions: Room temperature (22 ± 2 °C), relative humidity 40–50%; 24-hour curing period before testing.

3. **Experimental Setup and Testing Protocol**
   - Testing machine: Instron™ 8874 bi-axial servo-hydraulic testing machine (Instron, Norwood, MA, USA).
   - Compression plate: 150 mm diameter.
   - Loading rate: 5 mm/min axial displacement until failure or predefined endpoint.
   - Data acquisition software: testXpert III (ZwickRoell GmbH).

Table S1. Summary of Materials

Item	Manufacturer	Product Name/Model	Specifications	Catalog/Lot No.
PU Foam Block	Sawbones (USA)	Rigid PU Foam	ASTM Grade 20, 0.35 g/cm^3	1522-05
Femoral Stem	Zimmer Biomet	Metabloc Stem	126 mm, polished	—
Bone Cement	Heraeus Medical	Palacos® R+G	40 g powder, gentamicin-loaded	347621A
Cement Gun	Zimmer Biomet	Cement Gun 2	—	—
Cement Mixer	Heraeus Medical	VACUUM MIX 1	Vacuum 650 mmHg	—
Drill Bit	Dormer Tools	Carbide Twist Drill	8.5 mm	—
Testing Machine	Instron	Instron™ 8874	Bi-axial servo-hydraulic	—
Data Software	ZwickRoell	testXpert III	—	—
Alignment Jig	Custom	15° Wedge	15° fixed angle	—
Silicone Tubing	B. Braun	Sterile Silicone Tube	Ø 4 mm	—

Notes:
All experiments were performed under controlled laboratory conditions. The above protocols ensure reproducibility and compliance with ASTM F1839, ISO 7206-4, and ISO 7206-6 standards.
Please contact the corresponding author for additional technical details if required.
