# Supplementary material for: Biomechanical Impact of Cementation Technique Variations on Femoral Stem Stability: An In Vitro Polyurethane Model Study
Source: J Clin Med. 2025 May 8;14(10):3291. doi: 10.3390/jcm14103291 (PMC12112234; doi:10.3390/jcm14103291)
Supplement: Supplementary file 1 [file jcm-14-03291-s001.zip › File S2 - Checklist for Reporting In-vitro Studies (CRIS).pdf]

Supplementary File S2. Checklist for Reporting In-vitro Studies (CRIS)

Section/Topic	Item No.	Checklist Item	Reported on Page No.
Title and Abstract	1a	Identification as an in-vitro study in the title	1
Title and Abstract	1b	Structured abstract	1
Introduction	2a	Scientific background and rationale	2
Introduction	2b	Specific objectives	2
Methods	3	Intervention details	3–4
Methods	4	Defined outcomes	4
Methods	5	Sample size determination	3–4
Methods	6-8	Randomization, allocation, blinding	N/A
Methods	9	Statistical methods	5–7
Results	10	Numbers analyzed	8
Results	11	Results per group, effect sizes	8–11
Discussion	12	Interpretation	12–13
Discussion	13	Generalisability	13
Discussion	14	Trial limitations	14-15
Other Info	15	Registration, protocol access	N/A
Other Info	16	Funding	15
Other Info	17	Ethical approval	15
